# Chatbot-based health interventions in low- and middle-income countries: effective access, early attrition, and design strategies from a mixed-methods study

**DOI:** 10.1038/s44482-026-00027-5

**Published:** 2026-07-21

**Authors:** Max C. Klapow, Paula Zinser, Chiara Facciola, Qing Han, Maria da Graça Ambrósio, Francisco Antonio Calderón Alfaro, Bernice Mamauag, Jennel Reyes, Camille Habacon, Liane Peña Alampay, Samantha Erika Mendez, Rumaya Binti Juhari, Zainal Madon, Farah Zeehan Mohd Nadzri, Moa Schafer, Lily Clements, David Stern, Jamie M. Lachman

**Affiliations:** 1 Department of Experimental Psychology, University of Oxford, Oxford, UK; 2 Department of Social Policy and Intervention, University of Oxford, Oxford, UK; 3 IDEMS International, London, UK; 4 Institute of Population Research, Peking University, Beijing, China; 5 University of the Philippines Visayas, Miagao, Philippines; 6 Ateneo de Manila University, Quezon City, Philippines; 7 University of the Philippines Diliman, Quezon City, Philippines; 8 Universiti Putra Malaysia, Seri Kembangan, Malaysia

**Keywords:** Health care, Medical research, Scientific community

## Abstract

Equity in digital health is commonly assessed through enrolment and coverage. For interventions requiring sustained participation, enrolment can substantially overstate access. We report a convergent mixed-methods optimisation study of a chatbot-based behaviour change intervention for caregivers (ParentText) delivered via WhatsApp and Facebook Messenger across three low- and middle-income countries: Jamaica, Malaysia, and the Philippines (*N* = 1293). Using engagement logs, participant and implementer interviews, and a structured consensus process, we examined attrition patterns and explored optimisations. Median engagement was two days and one module; 93.8% of participants completed less than 25% of available content over the five-week intervention. Fewer than one in five participants remained active at Day 7 in two of three sites. Structural barriers included platform policies restricting follow-up after non-response, high message burden, and onboarding design. A consensus panel identified 23 barriers and generated 29 optimisations (69% rated highly feasible within LMIC contexts), currently being tested in a subsequent trial. We introduce *effective access* as a complementary construct for digital health interventions requiring repeated participation and propose three design principles: 1) test whether users can resume participation under platform constraints; 2) reduce message burden without removing content; and 3) treat re-engagement as a core feature.

## Introduction

Digital health interventions are increasingly deployed in low- and middle-income countries (LMICs) to expand access to evidence-based support, with equity often assessed using reach metrics such as enrolment and coverage^1,2^. However, enrolment does not guarantee sustained participation nor meaningful exposure to intervention content. For interventions designed to deliver support over multiple days or weeks, sustained engagement represents an important and under-examined dimension of access in digital health settings^3,4^. Prior expert consensus around engagement and attrition in digital mental health interventions has called for rigorous exploration of barriers to engagement and understanding how engagement affects optimal dosage^5^. The gap between who an intervention can initially reach and those who receive enough of it to benefit limits efforts to improve digital health equity, because dropout determines who continues to receive support and who does not.

In the present context of family wellbeing, equity concerns are particularly important for the populations these interventions are designed to serve. In LMICs, caregivers in low-income households, those with lower digital literacy, those with intermittent connectivity or shared devices, and those managing competing caregiving and work demands are primary targets of parenting support interventions and those for whom sustained self-directed digital engagement is most demanding^6,7^. These groups face elevated rates of child maltreatment risk and adverse developmental outcomes as well as greater risk of mental health problems^8^. These outcomes are compounded by stressors including social isolation, limited access to professional support, and poverty^9^.

In this paper, we propose *effective access* as a complementary evaluative construct for digital interventions that require repeated participation over time. We define it as the realised opportunity for meaningful intervention exposure, distinguishing it from enrolment (potential access) and from general engagement, retention, and completion. Engagement refers broadly to participant interaction with the intervention; retention to continued participation over time; completion to the proportion of prescribed content completed. In contrast, effective access refers to whether participants remain involved long enough to receive more than nominal exposure to the intervention as designed. This distinction matters most when people stop using a digital intervention because of the barriers they face, not because they chose to stop. Because no minimum effective dose has been established for ParentText, we measure effective access in this study using observable indicators of exposure, described in the Methods.

In-person delivery of parenting programmes, while effective, faces practical limits to scale in LMICs. Facilitator training, venue costs, and regular attendance place significant demands on users with limited resources^10,11^. Chatbot delivery via widely used messaging platforms offers a route to reaching substantially more people without proportional increases in cost or infrastructure^1^. However, this scalability depends on whether users can sustain participation over time.

Many LMIC digital health programmes are adapted from effective in-person interventions and delivered through automated chatbots to maximise scalability and minimise delivery costs^12–14^. Translating human-led programmes into self-directed automated interventions can introduce design challenges with implications for equity. In-person delivery typically offers provider or facilitator guidance, social reinforcement, flexible pacing, and structured opportunities to pause and resume care. In contrast, automated chatbot delivery requires users to navigate content independently and maintain attention under competing daily demands. For caregivers facing time scarcity and routine disruption, these structural differences may create systematic barriers to continued participation. In addition, the messaging platforms commonly used for LMIC chatbot delivery (e.g. SMS, WhatsApp, and Facebook Messenger) impose constraints due to usage policies and technical limitations that fundamentally shape how, when, and whether users can be re-contacted after non-response^15^. How these platform-level constraints interact with intervention design to influence sustained exposure across LMIC contexts remains under-examined.

Multi-country engagement datasets offer a practical opportunity to assess whether early attrition primarily reflects intervention or platform design features versus site-specific implementation factors. Such datasets are uncommon in LMIC digital health research due to heterogeneity in delivery systems, data capture, and the costs of multi-country partnerships. In this study, we analysed engagement patterns and barriers across three implementations of a chatbot-based preventive health intervention to reduce child maltreatment and promote healthy developmental outcomes delivered in Jamaica, Malaysia, and the Philippines (*N* = 1293). The intervention was adapted from Parenting for Lifelong Health’s in-person effective family health programmes for parents of young children and adolescents and delivered via automated messaging on SMS, WhatsApp, and Facebook Messenger^16,17^.

We conducted a convergent, mixed-methods optimisation study that integrated chatbot engagement logs, participant interviews, and a structured stakeholder consensus process using the nominal group technique^18^. Our aims were to (1) characterise engagement patterns relevant to equity in sustained exposure; (2) identify barriers to continued participation that were modifiable within platform and resource constraints; and (3) generate an optimised intervention intended to improve continuity and effective access in resource-limited settings. Using data from three LMIC settings, we examine how competing time demands, disruptions to daily routine, and platform policies may limit how long participants remain engaged with a digital intervention and we propose pragmatic design strategies to improve access for digital health interventions delivered at scale.

## Results

### Participant characteristics

A total of 1293 participants enroled across the three pilot sites: Jamaica (*n* = 1114), Malaysia (*n* = 82), and the Philippines (*n* = 97). The mean age across sites was 35.08. Most participants identified as women (80.74%). Participants selected content based on child age group: 40.29% enroled in the 2–9-year track, 39.83% in the 10–17-year track, and 13.15% in the 0–23-month track. Unless otherwise noted, descriptive engagement results reflect all enroled participants (*N* = 1293), whereas regression analyses reflect the analytic sample after pre-specified exclusions and missing-data handling (see ‘Methods’). Table 1 presents participant characteristics by country, including data completeness by site.

**Table 1:** Sample characteristics by country

Characteristic	Jamaica (*N* = 1114)	Malaysia (*N* = 82)	Philippines (*N* = 97)
Child age group, *n* (%)			
0–2 years	146 (13.1)	16 (19.5)	8 (8.2)
2–9 years	456 (40.9)	33 (40.2)	32 (33.0)
10–17 years	445 (39.9)	24 (29.3)	46 (47.4)
Did not answer	67 (6.0)	9 (11.0)	11 (11.3)
Child sex, *n* (%)			
Boy	122 (11.0)	20 (24.4)	18 (18.6)
Girl	80 (7.2)	24 (29.3)	9 (9.3)
Did not answer	912 (81.9)	38 (46.3)	70 (72.2)
Parent sex, *n* (%)			
Female	925 (83.0)	47 (57.3)	72 (74.2)
Male	99 (8.9)	17 (20.7)	21 (21.6)
Non-binary	23 (2.1)	—	—
Did not answer	67 (6.0)	18 (22.0)	4 (4.1)
Relationship status, *n* (%)			
Partnered	330 (29.6)	35 (42.7)	19 (19.6)
Single	182 (16.3)	—	9 (9.3)
Divorced	36 (3.2)	—	5 (5.2)
Widowed	7 (0.6)	—	1 (1.0)
Did not answer	559 (50.2)	47 (56.1)	63 (64.9)
Parent age			
Mean (SD)	34.98 (10.19)	36.57 (5.98)	34.99 (10.19)
Median	34.00	37.00	34.00

Dashes indicate data not collected at that site.

### Engagement patterns relevant to digital access and sustained use

Participants remained engaged for a short period relative to the planned 38-day intervention. Across the pooled sample, mean time in programme was 4.99 days (median: 2 days), and participants completed a mean of 1.35 modules (median: 1 module). Mean engagement duration varied by country (Philippines: 3.45 days; Jamaica: 4.69 days; Malaysia: 11.18 days). Across sites, 93.8% of participants completed less than 25% of the available intervention content, which we interpret descriptively as an indicator of minimal exposure to the intended intervention. Engagement distributions showed pronounced positive skew across sites with most participants disengaging within the first days and a small minority continuing substantially longer.

**Fig. 1:**
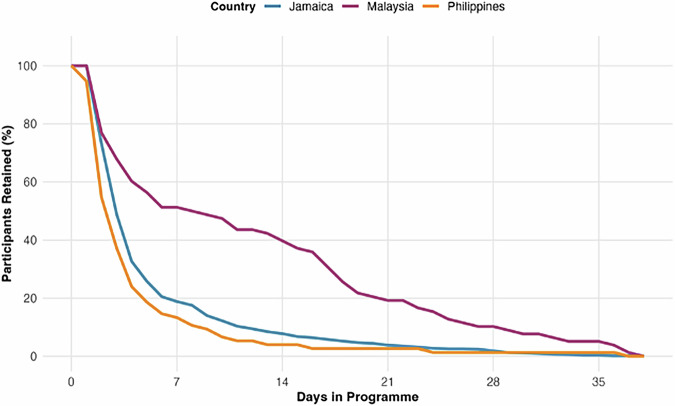
Participant retention over time by country. Retention curves show the percentage of enroled participants with at least one recorded inbound interaction at each day of the programme, by country. Jamaica: *n* = 1114; Malaysia: *n* = 82; Philippines: *n* = 97. Day 0 reflects onboarding. The planned intervention duration was 38 days. The Philippines reported that 5.3% of participants did not complete onboarding and are excluded from Day 0 onwards. Figure created using *R* (version 3.5.0).

Retention analyses indicated steep early attrition between onboarding (Day 0) and Day 4, with most disengagement occurring within the first week. Table 2 and Fig. 1 present retention at key timepoints across the studies. By Day 3, retention had declined to 49.0% in Jamaica, 67.9% in Malaysia, and 37.3% in the Philippines. By Day 7, fewer than one in five participants remained active in Jamaica (18.9%) and the Philippines (13.3%), while Malaysia showed higher sustained engagement (51.3%).

**Table 2:** Participant retention at key timepoints by country

Days in programme	Jamaica (%)	Malaysia (%)	Philippines (%)
0 (Onboarding)	100.0	100.0	94.7
3	49.0	67.9	37.3
7	18.9	51.3	13.3
14	7.8	39.7	4.0
30	1.2	7.7	1.3

Retention values correspond to the retention curves for Jamaica, Malaysia, and the Philippines at the listed days in programme (percent retained). Philippines reported that 5.3% of participants did not complete onboarding.

### Developmental stage and early dropout

Logistic regression examined whether early dropout varied by child developmental stage. Using the infant track (0–23 months) as the reference group, parents enroled in tracks for older children demonstrated significantly higher odds of dropout by Day 7. Parents in the child track (2–9 years) showed 2.24-fold higher odds of Day 7 dropout (OR = 2.24, 95% CI [1.47, 3.42], *p* < 0.001), and parents in the teen track (10–17 years) showed 1.78-fold higher odds (OR = 1.78, 95% CI [1.17, 2.67], *p* = 0.006).

### Module completion

Participants completed a mean of 1.59 modules overall, with differences by site: Jamaica (mean 1.34), Malaysia (mean = 4.04), and the Philippines (mean = 2.40).

Length in programme and modules completed were positively correlated within each site, although the strength of the association varied (Jamaica *r* = 0.33; Malaysia *r* = 0.63; Philippines *r* = 0.70), indicating that time in programme did not map uniformly onto progression through intervention content across settings (Table 3).

**Table 3:** Engagement summary statistics by country

Country	Variable	Mean	SD	Range	Correlation (95% CI)
Jamaica	Length in programme (days)	4.69	6.12	38.00	0.33***
	Number of modules completed	1.34	1.76	13.00	(0.28, 0.38)
Malaysia	Length in programme (days)	11.18	10.66	36.00	0.63***
	Number of modules completed	4.04	4.52	21.00	(0.48, 0.75)
Philippines	Length in programme (days)	3.45	5.29	36.00	0.70***
	Number of modules completed	2.40	4.27	23.00	(0.59, 0.79)

Correlation values represent the relationship between length in programme and number of modules completed within each country. Maximum possible modules ranged from 19 (infant age track) to 24 (teen track).

*SD* standard deviation, *CI* confidence interval.

***p < 0.001.

### Interaction patterns by message type

Interaction frequency varied by message type across sites. Among participants who were active, supportive messages offering additional content or resources (‘Supportive, Other’) generated the highest mean interaction rates in each country (Jamaica: 0.88; Malaysia: 4.28; Philippines: 2.63 interactions per active participant). Content messages also generated relatively high interaction rates (Jamaica: 0.57; Malaysia: 2.94; Philippines: 1.31). In contrast, standalone supportive praise messages generated the lowest interaction rates across all sites (Jamaica: 0.07; Malaysia: 0.95; Philippines: 0.60), a pattern referenced in the barrier synthesis below.

### Panel consensus on barriers to sustained engagement

The expert panel (*n* = 6) reviewed harmonised quantitative engagement summaries alongside translated qualitative excerpts and identified five primary barrier themes, comprising 23 specific challenges. Each barrier was assigned a malleability score (1–3; 1 = low, 3 = high), reflecting the extent to which the panel judged the barrier addressable within the scope of intervention modification.

### Structural features contributing to attrition (8 challenges; mean malleability = 2.63)

Across sites, the panel attributed early disengagement to a mismatch between programme structure and caregivers’ constrained time and external demands. The intervention’s 38-day delivery period exceeded the observed engagement window, with usage patterns indicating steep attrition after the first days. Qualitative excerpts were consistent with a time-burden mechanism:

*“It is unavoidable that I have not finished the programme yet. I cannot find the time for it due to my job.” (Participant 2, Malaysia)*

The panel identified message burden as a plausible contributor: three messages per day with individual messages often comprising multiple bullet points consolidated into long text blocks. Participants described frequency and length of messages as overwhelming:

*“The messages come too frequently for parents and they have a difficult time focusing on the programme because it can be overwhelming.” (Implementer, Jamaica)*

The time-bound progression system was also highlighted as a structural barrier. The chatbot advanced to new content daily regardless of whether participants had completed prior content, increasing the perceived cost of falling behind. In addition, messaging-platform rules restricting chatbot messages after non-response limited re-engagement when participants missed response windows. Participants described loss of conversational continuity (e.g. delayed replies triggering misunderstanding or restart) as frustrating and demotivating:

*“For example, you cannot send a reply right away because you’re cooking. When I send a reply later, it tells me that it does not understand what I said.” (Participant 7, Philippines)*

Additional structural challenges included insufficient support for re-entry after drop-off, perceived repetitiveness of messages, and low engagement with praise-related messages, consistent with the message type interaction pattern described above.

### Difficulty of use (7 challenges; mean malleability = 2.71)

The panel rated usability barriers as highly malleable, spanning both navigation friction and communication failures. Participants reported that the chatbot required precise inputs and did not robustly accommodate alternative phrasing, misspellings, or unanticipated responses:

*“It seemed that you really need to copy the words that the program uses… if you were not able to type in a word with an apostrophe, the program sends a reply…” (Participant 5, Philippines)*

The onboarding experience (‘Welcome Day’) was described as confusing, and in Jamaica some participants reportedly did not recognise the chatbot as automated. The help menu was perceived as difficult to navigate. Participants also expressed difficulty contextualising content framed as brief “tips” when they expected step-by-step skill-building:

*“The only thing I don’t get is the message on what to do when a child throws a tantrum…” (Participant 4, Philippines)*

Finally, participants reported limited ability to access or select specific content of immediate relevance, constraining perceived control over the intervention experience.

### Lack of engaging multimedia (3 challenges; mean malleability = 2.33)

Multimedia-related barriers were less prominent but highlighted accessibility and engagement trade-offs. Participants described audio and video content as too long or too dense to sustain attention. The panel noted that multimedia may be particularly relevant for accessibility, including potential benefits for users with literacy challenges, but noted that existing media did not optimally meet that need:

*“Video and voice notes are really helpful for parents, especially those with literacy challenges.” (Implementer, Jamaica)*

*“More variety. When you try several times, the [content] is the same.” (Participant 9, Malaysia)*

The panel also noted concerns about the cultural appropriateness and engagement value of amorphous figures used in videos and comics and identified perceived limitations in production quality.

### Few mechanisms to promote re-engagement (3 challenges; mean malleability = 2.67)

The panel identified limited support for recovery after interruption (pausing, falling behind, or routine disruption) as a central continuity barrier under messaging-platform constraints. The combination of (i) time-bound progression and (ii) restricted outbound follow-up after non-response was viewed as particularly challenging for caregivers whose routines made immediate responses difficult. Participants described difficulties resuming once a response window was missed:

*“Sometimes… I cannot answer right away… When this happens, I need to go back to the beginning because the program does not understand my answer.” (Participant 5, Philippines)*

Participants also reported that the chatbot timed out quickly (approximately 15–20 min), limiting the ability to pause and resume within a module:

*“Ever since my routine changed, I have not been on track and I needed to go back to the start more often.” (Participant 8, Philippines)*

### Lack of personalisation features (2 challenges; mean malleability = 2.50)

The panel identified personalisation barriers related to fit with household structure and misalignment between preset message delivery times and participants’ routines. First, caregivers with multiple children described limitations in tailoring content across children or switching between developmental tracks. Second, message delivery times were fixed rather than set by the user, contributing to missed prompts and disengagement during busy periods:

*“In the morning, when we are rushing…we just ignore it.” (Participant 9, Malaysia)*

Participants expressed interest in greater flexibility to catch up after delays:

*“…extend the period a little so that… the user will feel more comfortable and better able to catch up…” (Participant 4, Malaysia)*

Table 4 presents the barriers to engagement identified by the panel.

**Table 4:** Barriers to engagement identified through nominal group technique

Theme	Identified barriers	Number of challenges	Mean malleability score
Structural features contributing to attrition	• Intervention lasts too long • Significant dropout after Day 2 • Too many messages sent per day • Individual messages are too long • Praise messages are not engaging for parents • Messages are repetitive • Participants not progressing in timeline set by intervention • Insufficient messaging to continue participation in intervention	8	2.63
Difficulty of use	• Chatbot is difficult to navigate and understand limits of features • Help menu is confusing • Difficult to select specific content of interest • Welcome day and in-programme onboarding is confusing • Chatbot cannot handle responses that use alternative wording • “Tips” are hard to contextualise • Participants send messages that chatbot cannot understand	7	2.71
Lack of engaging multimedia	• Amorphous figures used as characters in media are not engaging and sometimes not appropriate for some cultural contexts • Video and audio messages are too long • Video quality needs refinement	3	2.33
Few mechanisms to promote re-engagement	• 24-h messaging limit promotes drop-out without reminder • Chatbot progress is time-bound • Chatbot times out quickly	3	2.67
Lack of personalisation features	• Chatbot cannot support multiple children simultaneously • Message delivery timing is not optimal for participants	2	2.50

Theme counts reflect the number of distinct challenges identified within each theme (not the prevalence of barriers among participants). Malleability scored on 1–3 scale: 1 = Low malleability (difficult to change within scope of optimisation), 3 = High malleability (feasible to change with available resources).

### Stakeholder Consensus on Optimisations (Modified Nominal Group Technique Stage 2)

**Fig. 2:**
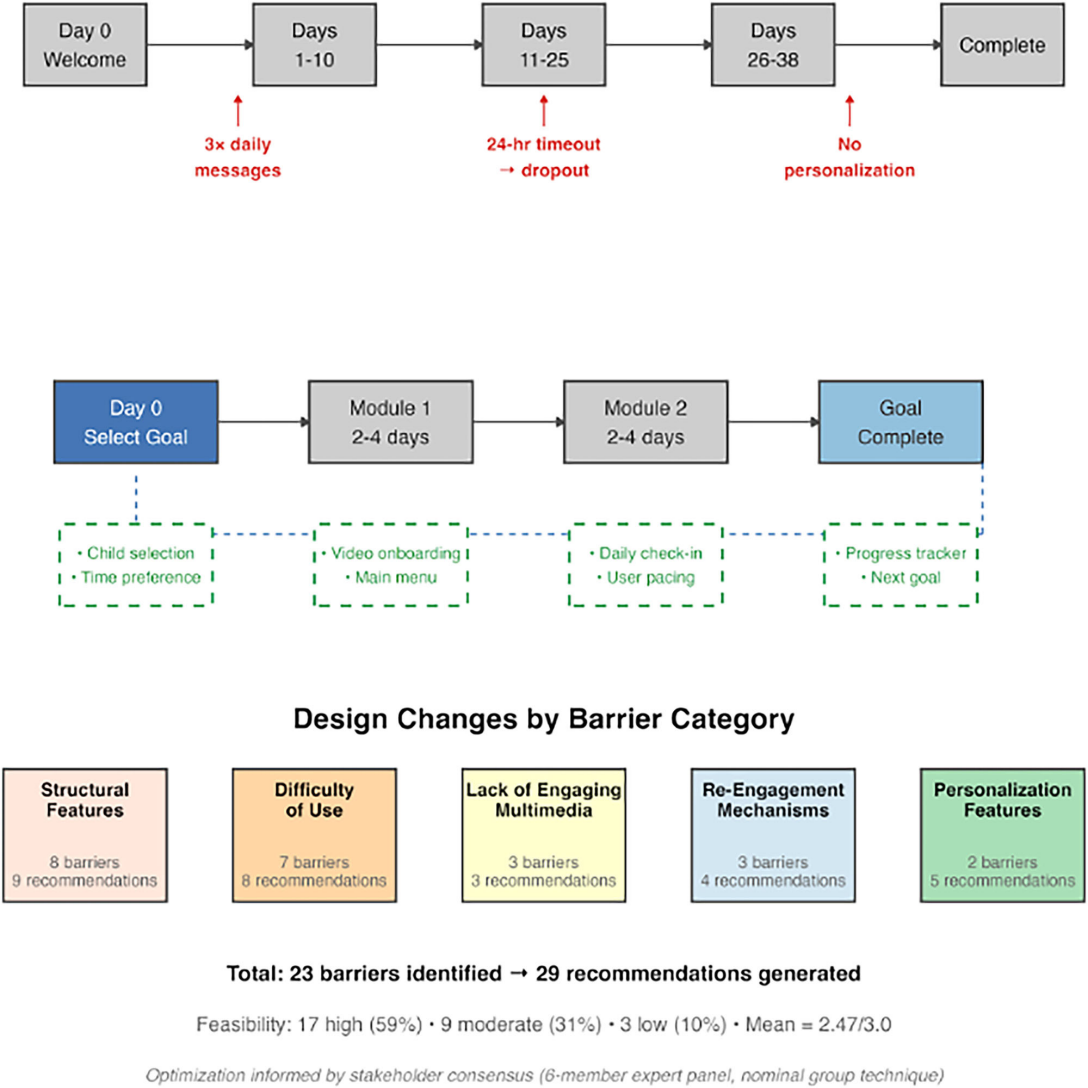
Original and optimised ParentText intervention structures. The top panel depicts the original intervention structure: a 38-day programme with three daily messages, time-bound sequential content progression, and a fixed 24-h re-engagement window governed by WhatsApp and Facebook Messenger automation policies. The bottom panel depicts the optimised structure generated through the modified nominal group technique consensus process, incorporating goal-oriented content series (2–4 days each), reduced messaging frequency (one interaction per day), progress-based rather than time-based advancement, and templated re-engagement reminders compatible with platform messaging policies. Figure created using *R* (version 3.5.0).

Using the same structured consensus method, the panel generated 29 optimisations addressing the barriers identified in Stage 1, described in Table 5. Optimisations were rated for feasibility (1–3; 1 = low, 3 = high). The mean feasibility score across optimisations was 2.59; 20 optimisations (69%) were rated high feasibility, six (21%) moderate feasibility, and three (10%) low feasibility. Figure 2 compares the original intervention structure to the optimised intervention structure.

**Table 5:** Optimisation by Barrier Category

Barrier theme	Specific challenges	Optimisations	Feasibility rating
Structural limitations	Significant dropout on and after Day 2	• Rework and simplify Welcome Day (Day 1) • Break down content on Day 2; integrate survey into modules	3 3
	Programme is too long	• Divide intervention into shorter goal-oriented content series (2–4 days long)	3
	Too many messages	• Reduce frequency of messages from 3 interactions/day to 1 interaction/day	3
	Messages are too long	• Shorten long messages by dividing into sequential parts	3
	Praise messages not engaging	• Delete standalone praise messages	3
	Messages predictable, less engaging	• Incorporate de-stressing and content-related exercises into daily messages	3
	Users not progressing in set timeline	• Remove timer mechanism; allow users to progress at own pace	3
	Difficult to re-engage after first week	• Use end of goal-oriented series as opportunity to start new goal	3
Difficulty of use	Difficult to navigate and understand features	• Send video or manual at end of first day introducing capabilities and limitations	2
	“Help menu” is confusing	• Redesign “help menu” into “main menu” with all settings and content access • Create troubleshooting flow for chatbot use issues	3 2
	Inability to choose content	• Allow users to choose content to access next within goal or from list of goals	2
	Welcome day too long	• Simplify welcome day by incorporating surveying later within modules	3
	Users send messages chatbot cannot understand	• Incorporate natural language processing	1
	Chatbot misunderstands when exact language not matched	• Make programme less sensitive to spelling mistakes; include local words and slang	3
	‘Tips’ are confusing terminology	• Change ‘tips’ to ‘tools’ or ‘skills’; consider incentives (badges, tokens)	3
Lack of engaging multimedia	Amorphous characters not engaging or culturally appropriate	• Replace with human figures and localised images	2
	Video and audio messages too long	• Shorten video and audio messages	1
	Video quality is low	• Hire professional agency to record video messages	1
Few mechanisms to promote re- engagement	24-h limitation causes dropout without reminder	• Utilise templated messages to send reminders exempt from 24-h limitation	3
	Users can’t find time for daily programme	• Change structure to sequential progress rather than time-based progress • Implement daily check-ins to identify progress and allow delays	3 3
	Chatbot times out quickly	• Allow more time to respond; consider removing timeout altogether	3
Lack of personalisation features	Cannot use programme for multiple children	• Allow users to choose which child to focus on at beginning of each goal • Allow users to swap between children and age groups • Ask questions before each module to customise to child’s name and age • Provide age-appropriate information if query about child submitted	2 2 3 3
	Timing of messages not optimal	• Allow users to choose time for message delivery	3

Feasibility rated on 1–3 scale: 1 = Low feasibility (substantial resource/technical constraints), 3 = High feasibility (implementable with existing resources).

### Structural features: reducing burden and aligning programme structure with observed engagement windows (mean feasibility = 2.78)

The panel reached consensus on fundamental programme restructuring to improve sustained use under real-world constraints. Rather than a single continuous 38-day programme, content was recommended to be reorganised into shorter goal-oriented series lasting 2–4 days. This structure was explicitly aligned with observed engagement patterns indicating most participants disengaged within the first week.

To reduce burden, the panel recommended reducing messaging frequency from three times daily to once daily and dividing long messages into sequential shorter segments. Standalone praise messages were recommended for removal. The panel also recommended shifting from time-bound to progress-bound advancement to allow participants to proceed at their own pace and resume without penalty after interruptions.

### Difficulty of use: onboarding, navigation, and communication robustness (mean feasibility = 2.45)

The panel prioritised onboarding and navigation redesigns intended to clarify the chatbot’s capabilities and reduce early friction. Recommendations included: simplifying ‘Welcome Day,‘ moving surveys into later modules to reduce initial burden, and providing an end-of-day video/manual explaining key features and limitations. The panel also recommended redesigning the help menu into a centrally accessible ‘main menu,’ adding a troubleshooting flow for common use issues, and enabling on-demand browsing of content goals.

To address communication failures, the panel recommended a low-feasibility option of incorporating natural language processing to handle varied phrasing, misspellings, and unanticipated inputs and identified a higher-feasibility alternative: expanding acceptable response lists to include common misspellings and local slang.

### Multimedia: accessibility benefits constrained by production feasibility (mean feasibility = 1.33)

Multimedia changes received the lowest feasibility ratings. The panel recommended replacing amorphous figures with localised human figures (moderate feasibility), shortening video/audio messages (low feasibility), and hiring professional agencies to improve quality (low feasibility).

### Re-engagement mechanisms under messaging-platform constraints (mean feasibility = 3.00)

Re-engagement optimisations received the highest feasibility ratings and directly targeted barriers linked to the WhatsApp/Facebook Messenger constraint and time-bound progression. The panel recommended: (1) using templated messages pre-approved by the messaging platform to deliver reminders exempt from the 24-h limitation; (2) implementing daily check-ins to determine progress and resume from the appropriate point rather than introducing new content; and (3) extending or, where possible, removing the chatbot timeout period to allow users to pause and resume within modules.

### Personalisation: improving fit to household structure and routines (mean feasibility = 2.60)

To support caregivers with multiple children, the panel recommended enabling child selection at the start of each goal-oriented series and incorporating brief personalisation questions to tailor content. To address misalignment with daily routines, the panel recommended allowing users to select preferred delivery times for reminder messages during onboarding.

## Discussion

This study addresses an important evaluation gap in digital health. For interventions intended to be used repeatedly, enrolment and coverage figures may substantially overstate how many people actually received the intervention. Using a multi-country dataset from three LMICs and a convergent mixed-methods optimisation approach, we provide cross-context evidence on early attrition in messaging-based chatbot delivery and translate that evidence into design priorities for scale. In total, we identified 23 barriers to sustained engagement with a digital preventive health chatbot intervention for families across three LMIC pilot implementations and generated 29 redesign optimisations through a structured stakeholder consensus process.

Across settings, most participants disengaged within the first week despite the programme being designed to last 38 days. Redesign recommendations primarily targeted structural features, including message burden, pacing, progression logic, onboarding, and re-engagement rather than core intervention content. The consensus panel rated 69% of optimisations as highly feasible given technical and resource constraints, reflecting a focus on changes that can be implemented at scale in LMIC settings. Methodologically, the modified nominal group technique provided a structured process for translating heterogeneous evidence into implementable redesign priorities, including explicit trade-offs noted via malleability and feasibility ratings. This approach may be especially useful in digital health settings where teams need to prioritise rapid adaptations under tight constraints. Selected optimisations were subsequently tested in randomised optimisation trials in South Africa and Malaysia^19,20^. South Africa was selected as the in-person programme’s country of origin, with established implementation partnerships and trial infrastructure; extension to Jamaica and the Philippines is planned contingent on funding.

Engagement patterns were similar across sites despite substantial differences in language, recruitment approach, and implementation context, indicating that there may be equity-related design constraints present across settings. The concentration of attrition in Days 0–7 suggests that early interaction and re-engagement mechanisms may be particularly consequential for equitable exposure in messaging-based chatbot interventions, though site-level factors could also contribute to this pattern. Additionally, qualitative data indicate that caregivers valued the intervention and its content and disengaged due to structural barriers inherent to the intervention’s design rather than a lack of perceived relevancy or interest.

Although the pattern of attrition was similar across countries, retention and completion levels differed by site (Fig. 1; Tables 2 and 3). By Day 7, retention was 51.3% in Malaysia versus 18.9% in Jamaica and 13.3% in the Philippines, alongside longer mean engagement duration (11.18 vs 4.69 vs. 3.45 days) and higher module completion (mean 4.04 vs. 1.34 vs. 2.40). These differences may reflect how each site was implemented, by government-partner recruitment and delivery in Malaysia, community recruitment in Jamaica, and recruitment of low-income families through a conditional cash transfer programme in the Philippines. Malaysia’s higher engagement may also reflect higher rates of connectivity and digital literacy^21,22^, though this is difficult to assess given the small sample and differences in implementation context. We therefore interpret cross-site similarities as evidence of shared design constraints, while recognising that implementation conditions may have influenced engagement levels at each site.

While selective or need-based use cannot be ruled out entirely, ParentText was designed as a structured programme to be completed over 5 weeks, not as an on-demand resource. The consistent pattern of early dropout across all three sites, and participant accounts describing practical barriers rather than lack of interest as reasons for stopping, suggest that most early attrition reflected barriers to continued use rather than selective engagement. Future work should test whether brief engagement is sufficient for benefit in similar programmes.

In digital health, equity is often measured by reach—who can download an app, enrol in a programme, or connect to a telehealth service^6^. Our findings support a complementary dimension: effective access, defined here as sustained participation sufficient to receive more than nominal exposure to an intervention designed for repeated use. This is consistent with frameworks that describe equity as shaped not only by whether someone can initially access a service, but also by whether they can continue using it^7^. Across three LMIC pilots, median engagement was two days and one module, and 93.8% of participants completed less than 25% of available content. These results show that enrolment can substantially overstate how many people actually received the intervention. Participant accounts indicate that caregivers who faced competing demands such as work, childcare, and household responsibilities found the intervention’s message frequency and pacing difficult to sustain, and disengaged early as a result.

The gap between enrolment and effective access matters most to improve digital health equity because the same features that make messaging-based chatbot interventions attractive at scale (e.g. low cost, wide reach, fewer developmental demands) also make structured intervention formats the hardest to sustain for people they are designed to reach. For digital interventions that require sustained use to be effective, dropout is therefore a fundamental equity concern, as it determines who actually receives the programme and who does not. This is likely most important for users with less time, less predictable daily routines, or fewer resources to manage competing demands. This study did not directly test whether dropout varied by socioeconomic or demographic characteristics, though future work should examine this directly. Further, these findings are consistent with prior calls to treat discontinuation and non-use as core outcomes in digital health evaluation rather than incidental user behaviour^5,23,24^.

A central finding of this study is that platform-level policies can directly limit how much of an intervention participants receive, in ways that are especially likely to affect users with less control over their time. The chatbot was delivered via the most widely used messaging platforms in each country because these channels offer broad reach and are feasible to implement at low cost in LMIC settings. The platforms’ automation policies, particularly those restricting re-contacting users who did not respond to an earlier automated message within a defined window, created an engagement pathway that quickly crumbled when participants could not respond immediately^25^. When this happened, participants often lost their place in the conversation, could not resume smoothly, or had to restart, which they described as confusing and demotivating.

This illustrates a broader point: digital platforms are not neutral components of implementation. The rules governing widely used messaging platforms directly affect whether users can re-engage after missing a message, and therefore how much of the intervention they receive. Our redesign responded by restructuring the intervention to work within these constraints by shifting to progress-based content delivery, adding re-engagement mechanisms, and using platform-approved templated messages to send reminders within allowable limits^26^.

Although this study focused on a parenting chatbot to reduce violence against children and parental stress, the engagement barriers observed across three LMIC pilots represent broader vulnerabilities in chatbot-delivered health interventions. Based on engagement logs, participant accounts, and the panel’s highest-feasibility optimisations, we propose three pragmatic design principles to support effective access.

First, test whether users can resume the intervention under the worst likely conditions. Many chatbot systems support smooth re-engagement in principle, but messaging-platform health interventions frequently operate under tighter constraints (session windows, restricted outbound follow-up, and rule-bound automation). Before deployment, check whether a user who returns after hours or days, with partial completion, can reliably continue without extra support and without the system violating platform rules. Recovery prompts should be clear, navigation should be simple, and re-engagement pathways should be designed with platform limitations in mind.

Second, reduce message burden without removing content. An intervention is not accessible if it requires more time and attention than participants can reliably provide. In this study, participants described the programme as overwhelming due to message frequency, message length, and total duration. The most feasible optimisations reduced burden by lowering message frequency, breaking long messages into shorter parts, and restructuring content into short series lasting two to four days, matched directly to the engagement window observed in the data. The goal is to make the delivery format workable for people with limited and unpredictable time while not meaningfully reducing the overall level of support.

Third, treat re-engagement as a core feature when navigating platform constraints. For chatbot interventions, re-engagement determines whether an interruption is a temporary pause or the end of participation entirely. The most feasible optimisations in this study directly targeted re-entry (e.g. templated reminders when allowed, daily check-ins, resumable flows). Sustained exposure depends on maintaining engagement under real-world conditions, which may require substantial redesign of intervention pacing, progression, and follow-up to fit platform rules and participants’ routines. We should not assume consistent adherence. Instead, we should design interventions so that someone who misses several days can return and continue without confusion or penalty. This matters most for users with unpredictable routines and competing demands, who are also those most likely to drop out when re-engagement is difficult.

These findings have practical implications for implementers and funders pursuing equitable digital health scale-up. First, platform selection should be treated as an equity decision with downstream consequences for continuity, re-engagement, and personalisation. Teams should evaluate not only reach and cost, but also automation policies and constraints that affect sustained engagement. Second, intervention structure should be designed around observed engagement capacity, using early dropout from formative evaluations as a signal to redesign intensity, pacing, and progression rather than attributing disengagement to motivation alone. Third, accessibility and localisation costs (including culturally appropriate media and robust translation) should be planned as core components of equitable delivery rather than optional enhancements.

A key strength of this work is the triangulation of engagement logs, qualitative interviews, and structured stakeholder consensus across three LMIC implementations, enabling identification of barriers that recurred despite differences in context and recruitment. We also note several limitations. First, optimisations were not experimentally evaluated in this study, thus, causal claims about their effects on engagement cannot be made. Second, interview data were available from two sites, and the Jamaica pilot contributed an implementer interview rather than participant interviews, which may limit representation of user perspectives. Third, while the panel included members who had completed the programme previously as participants, it did not include programme participants from the pilots themselves, which could have influenced prioritisation toward implementer- and developer-oriented solutions. The equity framing in this paper is structural and inferential rather than empirically demonstrated through participant-level stratification. We did not have sufficient demographic data across sites to test whether attrition varied by markers of disadvantage, such as income or digital literacy. Our argument is that the barriers to access identified in the study are likely to be most consequential for users with the fewest resources, which is consistent with the populations targeted by these pilots, though not directly tested. Future work should examine whether effective access varies by participant-level factors. Finally, findings reflect the constraints of the specific messaging platforms used; other platforms may present different trade-offs.

This work provides early evidence in LMICs that, for messaging-based interventions requiring repeated participation, equitable access cannot be inferred simply from enrolment. Across three pilots, most participants disengaged within the first week, showing how programmes can reach large numbers while failing to provide meaningful exposure to those facing the greatest constraints. Our convergent mixed-methods approach and structured consensus process translate these engagement inequities into implementable design principles. Equity-oriented chatbot design should assume discontinuation, treat re-entry pathways as a core feature, and invest in formative optimisation that better matches the structure of digital health interventions to the time and resource scarcity of intended users.

## Methods

### Study design and objectives

ParentText is a digital health intervention that delivers evidence-based violence prevention and parenting support to caregivers through automated messaging platforms. Following pilot implementation across three LMICs (Jamaica *n* = 1114; Malaysia *n* = 82; Philippines *n* = 97, total *n* = 1293), engagement data indicated substantial early attrition despite a rigorous content adaptation and stakeholder co-design process during development. We therefore conducted a convergent mixed-methods study to (i) identify digital intervention structure and user experience factors associated with low sustained engagement and (ii) generate evidence-informed, feasible optimisations through a structured stakeholder consensus process.

We analysed multi-country pilot data to explore whether early engagement barriers were consistent across diverse implementation contexts, and to prioritise redesign strategies that are feasible within common constraints of population-scale messaging-platform delivery in LMIC settings. The study was guided by the UK Medical Research Council (MRC) Framework for the Development and Evaluation of Complex Interventions (Skivington et al., 2021). To operationalise redesign decisions, we also drew on the Six Steps in Quality Intervention Design (6SQUID) Framework to operationalise the redesign process (Wight et al., 2016). Specifically, we used Steps 2–4 of 6SQUID to assess which barriers were within scope to modify, identify potential mechanisms, and select feasible delivery modifications.

### Ethics approval

This study was conducted in accordance with the Declaration of Helsinki. The study was approved by the University of Oxford Central University Research Ethics Committee (Ref: R69569/RE007), Ateneo de Manila University’s Research Ethics Committee (Ref: AdMUREC_21_088), and Universiti Putra Malaysia’s Ethics Committee for Research Involving Human Subjects (Ref: JKEUPM-2020-417). Principal investigators for the Jamaica, Malaysia, and Philippines pilot studies granted permission for secondary analyses of available engagement data. Informed consent was obtained from all participants by the principal investigators of the original pilot studies in accordance with local ethics committee requirements. The present study used anonymised engagement data provided under secondary data sharing agreements; no additional participant consent was required.

### Setting and participants

This study was conducted in collaboration with UNICEF as part of the implementation of the intervention across multiple LMIC settings. The primary objective was to characterise engagement under routine, naturalistic delivery conditions, with implementation led by in-country partners (government and/or local NGOs) using the RapidPro platform. Sites were selected pragmatically based on UNICEF country office engagement and operational feasibility, including capacity to host and deliver the programme via RapidPro and to support recruitment through established partnerships with government and local NGO stakeholders. Participants were not incentivised to complete modules, and engagement reflected participant-initiated use under standard delivery procedures.

Recruitment occurred between August 2021 and January 2022 and was conducted by UNICEF, NGO or government partners. In Jamaica (*n* = 1114), participants were recruited from four parishes in collaboration with UNICEF Jamaica. In Malaysia (*n* = 82), participants were employees and previous programme beneficiaries of the National Population and Family Development Board and were recruited by trainers within the same ministry. In the Philippines (*n* = 97), participants were low-income families enroled in a conditional cash transfer programme and were recruited in collaboration with the Department of Social Welfare and Development. Across sites, pilots initially aimed to recruit approximately 100 caregivers per country; however, available implementation resources varied by country, resulting in substantially larger recruitment in Jamaica due to additional local budget and delivery support. Given the resulting imbalance in sample size, descriptive results are reported by site rather than pooled across countries.

### Intervention

ParentText is derived from Parenting for Lifelong Health (PLH)’s in-person, group-based parenting programmes designed to reduce violence against children^16,17^. PLH programmes aim to improve positive parenting skills through psychoeducation and skill development across four themes: (1) praising the child, (2) increasing quality time with the child, (3) setting rules and responsibilities, and (4) coping with negative emotions and responding to crises. The in-person programme had been previously delivered in both Malaysia and the Philippines^27–29^.

ParentText delivers adapted content through automated messages to caregivers of children aged 0–17 years. The intervention was implemented via RapidPro, an open-source messaging platform developed by UNICEF, and delivered through WhatsApp, Facebook Messenger, and SMS. Platform selection imposed key design constraints. WhatsApp and Facebook Messenger Business APIs restrict automated messaging such that, if users do not respond, only one unprompted message may be sent within a 24-h period. This limitation constrained re-engagement capacity for participants who experienced interruptions and was therefore a central focus of the optimisation process.

Content was tailored to the child’s developmental stage (0–23 months, 2–9 years, 10–17 years) and emphasised three themes: (1) positive reinforcement, (2) building a positive caregiver–child relationship, and (3) reducing caregiver stress and improving coping skills. Content was pre-set based on child demographics. The intervention contained three tracks with similar content, delivered in identical structure, but adapted for three developmental stages: baby, child, and teen. Caregivers in each track had access to the same number of modules. Additionally, caregivers had 24/7 access to on-demand troubleshooting content via a ‘help’ trigger word. Completion rates accounted for the specific age-appropriate track caregivers completed. All content underwent adaptation before implementation to ensure cultural relevance and appropriate translation. The intervention was delivered over approximately 5 weeks (38 days). Messages were sent three times per day and varied between supportive messages, delivery of new content, including practice exercises, and check-ins. Participants progressed through content using a time-based schedule unless they disengaged. The intervention delivered content sequentially over the course of the five-week intervention. Participants were encouraged to complete all modules during the study. These conditions reflect routine, naturalistic implementation; participants were not incentivised to complete modules and no additional researcher contact was used to prompt adherence.

Cultural adaptation was undertaken as a pragmatic surface adaptation process in partnership with UNICEF country offices, local NGO/government implementation partners, and caregivers. This process focused on contextualising programme content for local delivery (including review of examples, terminology and delivery scripts), translation and back-translation where relevant, and iterative user testing prior to deployment to ensure clarity, acceptability and feasibility within implementation constraints. This pragmatic adaptation approach was used to enable timely delivery; insights from these pilots subsequently informed more extensive co-design and refinement work reported elsewhere^19,30–32^.

### Quantitative data: collection and analysis

Engagement data were recorded automatically by UNICEF RapidPro servers, exported at the end of each pilot, anonymized, and transferred to the University of Oxford secure server. Analyses were restricted to variables available across all sites: demographic information, length of time spent in the programme (days between first interaction and last recorded interaction), number of modules completed, and number of interactions by message type. Throughout this paper, we use ‘engagement’ to refer to the overall extent of participant interaction with the intervention, ‘retention’ to describe continued participation at successive timepoints, and ‘completion’ to describe the proportion of available modules completed. Where the emphasis is on receiving sufficient intervention content to plausibly benefit, we use the term *effective access*. In this study, effective access was operationalized provisionally using observable proxies to exposure, as no validated minimum effective dose has been established for ParentText. The indicators used were time in programme, days active, modules completed, and the proportion of participants completing less than 25% of available content. The <25% threshold was selected as a deliberately conservative lower bound to identify participants who received only a small minority of available content in an intervention explicitly designed for structured delivery over 38 days. This threshold is used as a descriptive indicator of minimal exposure, not as a validated efficacy cutoff. We note that the extreme positive skew of the engagement distribution means this finding is robust to the specific threshold chosen; sensitivity analyses using alternative cutpoints are reported in Supplementary Information.

All analyses were conducted in R (version 3.5.0). We summarised engagement using descriptive statistics and visualisations. Completion rates were calculated as completed modules divided by the total available modules for a participant’s selected child age track. To quantify the extent of participant exposure to intervention content, we calculated pooled median days in programme and median modules completed across all sites. A participant was considered active on a given day if RapidPro recorded at least one inbound interaction (button selection or free-text response) that day. Days active was calculated as the sum of the number of days on which a participant was active. We also calculated the percentage of participants completing less than 25% of available content (defined as <5 modules for the infant track, <6 modules for the child and adolescent tracks) as an indicator of minimal intervention exposure. Time in the programme was defined as the number of days between a participant’s first recorded interaction and last recorded interaction in RapidPro. This measure captures the span of observed interaction but does not distinguish continuous participation from late re-contact after a period of inactivity. Because ParentText was designed as a 38-day programme, primary analyses characterising exposure to the intended intervention dose were restricted to participants whose recorded time in the programme was ≤38 days. We conducted a sensitivity analysis retaining all enroled participants but capping time in the programme at 38 days for retention calculations (Supplementary Information), to assess whether conclusions about early attrition and minimal exposure were robust to late re-contact outside the planned intervention window.

To examine whether early dropout varied systematically by child age group, logistic regression was conducted predicting dropout by Day 7. Child age group (infant: 0–23 months; child: 2–9 years; teen: 10–17 years) was included as a predictor, with infant track as the reference category. Analysis was conducted on the combined dataset (*N* = 1166) after excluding participants with engagement >38 days (the planned intervention length; *n* = 1089 after listwise deletion of missing predictors; see Supplementary Information). All tests were two-tailed with *α* = 0.05. Quantitative results were summarised as briefing materials to inform the structured stakeholder consensus process described below.

### Collection and synthesis of qualitative data for secondary analysis

Two pilot sites conducted qualitative interviews to understand barriers and facilitators to use and the acceptability of chatbot content. In the Philippines, nine participants were interviewed in Filipino by local researchers (approximately 60 min). In Malaysia, nine participants (34.6% response rate from 26 contacted) were interviewed in Malay by officers of the National Population and Family Development Board. Interviews were transcribed and analysed using thematic analysis^33^ by two coders. Sampling was guided by participant availability and the principle of information power, prioritising depth and richness of data over the number of interviewees^34^. Participants were sampled by availability in the Philippines and Malaysia. To supplement participant interview data, an additional 45-min interview was conducted within the present study with the lead implementer from the Jamaica pilot. The convergent mixed-methods approach allowed the consensus panel (described below) to first describe engagement patterns based on pooled pilot data, then consider participant and implementer qualitative data to provide additional context^35^.

### Stakeholder consensus process for optimisation: modified nominal group technique

During study conception, we specified that findings would inform an updated iteration constrained primarily to structural changes in chatbot functioning rather than major content changes unless deemed necessary. This focus is consistent with conceptual models of digital behaviour change interventions suggesting that engagement-related design elements (e.g. timing, frequency, goal-related messaging) may moderate intervention effects^36,37^.

After initial data aggregation, we selected a structured consensus approach rather than additional open-ended qualitative analysis because: (1) the dataset included multiple data modalities and was likely to yield conflicting evidence; (2) interpreting engagement barriers required technical and domain-specific expertise; and (3) there is limited published evidence on optimising parenting chatbots for LMIC delivery contexts. Consensus methods can synthesise complex or conflicting information that cannot be analysed quantitatively^38^.

We selected a modified nominal group technique (NGT) rather than a Delphi approach to enable structured discussion and idea generation, as anonymity was not required. NGT is a structured method that uses facilitated discussion and voting to generate and refine priorities and has been used to inform health intervention development and refinement and offers participants opportunities to brainstorm, discuss, and vote iteratively^39,40^. We conducted the NGT in two stages: identification and categorisation of engagement barriers (Stage 1) and feasibility assessment of proposed optimisations (Stage 2).

The panel comprised six stakeholders: a PLH program manager who did not participate in the pilot programmes, the ParentText project manager, a master trainer for the in-person PLH programme, a technical specialist from IDEMS International who led key technological aspects of the chatbot, a researcher specialising in engagement with digital interventions, and a researcher specialising in intimate partner violence in parenting programmes (*n* = 6). The first author facilitated the group. All panellists had direct experience implementing digital health interventions in LMIC settings. Some panellists had lived experience as caregivers and prior users of the intervention, though caregivers from the three pilot sites were not included due to logistical constraints and to minimise confidentiality and coercion concerns.

Prior to Stage 1, panellists received supplementary materials including a problem statement (‘What are the reasons for low engagement with the intervention?’), pilot study descriptions, and data overviews^41^. The panel reviewed quantitative descriptive findings and qualitative excerpts and generated preliminary barrier categories using round-robin brainstorming recorded by the facilitator.

For iterative coding, each piece of qualitative data was discussed and voted into barrier categories. Voting proceeded using an 80% majority threshold. When disagreements occurred, additional round-robin discussion was conducted followed by a revote. This process was repeated for three rounds until all excerpts were categorised or excluded as not relevant.

After five categories were identified, the panel reconvened to review data placement. Given the exploratory nature of the study and potential cross-site variation, the panel did not rank the barriers; instead, we recorded the number of data excerpts contributing to each category. Malleability ratings were assigned by the panel following review of the quantitative engagement data and qualitative excerpts mapped to each barrier. Each panellist first proposed a rating (1 = low, 3 = high) based on the perceived scope to address the barrier through changes to chatbot structure, content presentation, or implementation procedures. Ratings were then discussed in round-robin format and finalised by vote using an 80% majority threshold; unresolved disagreements were revisited after further discussion and re-voted.

Using the same modified NGT process, the panel generated optimisations to address barriers identified in Stage 1. Barriers rated as moderate or high malleability were prioritised. Proposed solutions were presented round-robin and subjected to an initial vote on whether they should be discussed (80% threshold). For solutions taken forward, each panellist commented before a final vote on inclusion.

Included optimisations were then rated for feasibility on a 1–3 scale (1 = low feasibility, 3 = high feasibility), defined as the extent to which the recommendation could be implemented given time and resource requirements. The same discussion and voting protocol (80% threshold) were used. The facilitator summarised final challenges and proposed solutions for panel review; panellists provided further refinements during meetings and, if desired, by email afterward.

## Supplementary information

Supplementary information

## Data Availability

The datasets analysed during the current study were generated through pilot implementations of ParentText conducted in collaboration with UNICEF and in-country implementation partners. Due to ethical and governance restrictions and the potential identifiability of participants through trace data, the de-identified RapidPro engagement logs and qualitative transcripts are not publicly available. Access to a de-identified dataset and custom R scripts sufficient to reproduce the analyses reported in this manuscript may be made available from the corresponding author on reasonable request, subject to approval by relevant partner organisations and ethics requirements.
