# Supplementary material for: Chatbot-based health interventions in low- and middle-income countries: effective access, early attrition, and design strategies from a mixed-methods study
Source: NPJ Digit Public Health. 2026 Jul 21;1(1):23. doi: 10.1038/s44482-026-00027-5 (PMC13388102; doi:10.1038/s44482-026-00027-5)
Supplement: Supplementary file 1 — Supplementary information [file 44482_2026_27_MOESM1_ESM.pdf]

Supplementary Information: Sensitivity Analysis of Retention Estimates with Late Re-contact

MCK

2026-02-25

Overview and rationale

ParentText was designed as a structured programme delivered over 38 days. In the main manuscript, retention and engagement are analysed within this planned programme window to characterise exposure to the intended intervention dose.

In RapidPro, time in programme was operationalised as the number of days between a participant's first recorded interaction and last recorded interaction. This measure captures the span of observed interaction but does not distinguish continuous participation from late re-contact after a period of inactivity. As a result, participants who re-contact the chatbot after the planned programme period may appear "retained" at later timepoints even if they did not participate continuously in programme content.

This appendix reports (1) how often recorded engagement durations exceeded 38 days and (2) a sensitivity analysis assessing whether conclusions about early attrition within the planned window are robust when late re-contact is handled conservatively.

Extent of engagement durations >38 days

```
## # A tibble: 4 × 4
##   country         N n_over_38 pct_over_38
##   <chr>       <int>     <int>       <dbl>
## 1 Jamaica      1114       101        9.07
## 2 Malaysia       82         4        4.88
## 3 Philippines    97        22       22.7 
## 4 Pooled       1293       127        9.82
```

Supplementary Table 1. Participants with recorded engagement durations exceeding the planned 38-day programme window.

country	N	n_over_38	pct_over_38
Jamaica	1114	101	9.1
Malaysia	82	4	4.9
Philippines	97	22	22.7
Pooled	1293	127	9.8

```
## Across the pooled sample, 127/1293 participants (9.8%) had recorded engagement durations exceeding 38 days.
```

Sensitivity analysis approach

To assess whether conclusions about retention within the planned programme window were robust to late re-contact outside the intended 38-day period, we conducted a sensitivity analysis in which we retained all enrolled participants but capped time in programme at 38 days for retention calculations (i.e., time_in_programme_capped = min(time_in_programme, 38)). Retention was then recalculated as the proportion of participants with capped time in programme greater than or equal to each timepoint (Days 1, 3, 7, 14, and 30), using the original denominators for each site.

This approach tests robustness of conclusions about early attrition and exposure to the intended intervention dose, while preventing late re-contact from being misinterpreted as continuous programme retention. ### Primary analysis

Supplementary Table 2. Primary retention at key timepoints (restricted to participants with time in programme ≤38 days).

country	day	formatted
Jamaica	1	100.0% (n=1013/1013)
Jamaica	3	49.0% (n=496/1013)
Jamaica	7	18.9% (n=191/1013)
Jamaica	14	7.8% (n=79/1013)
Jamaica	30	1.2% (n=12/1013)
Malaysia	1	100.0% (n=78/78)
Malaysia	3	67.9% (n=53/78)
Malaysia	7	51.3% (n=40/78)
Malaysia	14	39.7% (n=31/78)
Malaysia	30	7.7% (n=6/78)
Philippines	1	94.7% (n=71/75)
Philippines	3	37.3% (n=28/75)
Philippines	7	13.3% (n=10/75)
Philippines	14	4.0% (n=3/75)
Philippines	30	1.3% (n=1/75)
Pooled	1	99.7% (n=1162/1166)
Pooled	3	49.5% (n=577/1166)
Pooled	7	20.7% (n=241/1166)
Pooled	14	9.7% (n=113/1166)
Pooled	30	1.6% (n=19/1166)

Supplementary Table 3. Sensitivity retention at key timepoints (all participants retained in denominators; time in programme capped at 38 days for retention calculations).

country	day	formatted
Jamaica	1	100.0% (n=1114/1114)
Jamaica	3	53.6% (n=597/1114)
Jamaica	7	26.2% (n=292/1114)
Jamaica	14	16.2% (n=180/1114)
Jamaica	30	10.1% (n=113/1114)
Malaysia	1	100.0% (n=82/82)
Malaysia	3	69.5% (n=57/82)
Malaysia	7	53.7% (n=44/82)
Malaysia	14	42.7% (n=35/82)
Malaysia	30	12.2% (n=10/82)
Philippines	1	95.9% (n=93/97)
Philippines	3	51.5% (n=50/97)
Philippines	7	33.0% (n=32/97)
Philippines	14	25.8% (n=25/97)
Philippines	30	23.7% (n=23/97)
Pooled	1	99.7% (n=1289/1293)
Pooled	3	54.4% (n=704/1293)
Pooled	7	28.5% (n=368/1293)
Pooled	14	18.6% (n=240/1293)
Pooled	30	11.3% (n=146/1293)

Comparison of primary vs sensitivity retention estimates

Supplementary Table 4. Comparison of primary retention estimates vs capped-duration sensitivity estimates (formatted as % retained with counts).

country	day	primary	capped
Jamaica	1	100.0% (n=1013/1013)	100.0% (n=1114/1114)
Jamaica	3	49.0% (n=496/1013)	53.6% (n=597/1114)
Jamaica	7	18.9% (n=191/1013)	26.2% (n=292/1114)
Jamaica	14	7.8% (n=79/1013)	16.2% (n=180/1114)
Jamaica	30	1.2% (n=12/1013)	10.1% (n=113/1114)
Malaysia	1	100.0% (n=78/78)	100.0% (n=82/82)
Malaysia	3	67.9% (n=53/78)	69.5% (n=57/82)
Malaysia	7	51.3% (n=40/78)	53.7% (n=44/82)
Malaysia	14	39.7% (n=31/78)	42.7% (n=35/82)
Malaysia	30	7.7% (n=6/78)	12.2% (n=10/82)
Philippines	1	94.7% (n=71/75)	95.9% (n=93/97)
Philippines	3	37.3% (n=28/75)	51.5% (n=50/97)
Philippines	7	13.3% (n=10/75)	33.0% (n=32/97)
Philippines	14	4.0% (n=3/75)	25.8% (n=25/97)
Philippines	30	1.3% (n=1/75)	23.7% (n=23/97)
Pooled	1	99.7% (n=1162/1166)	99.7% (n=1289/1293)
Pooled	3	49.5% (n=577/1166)	54.4% (n=704/1293)
Pooled	7	20.7% (n=241/1166)	28.5% (n=368/1293)
Pooled	14	9.7% (n=113/1166)	18.6% (n=240/1293)
Pooled	30	1.6% (n=19/1166)	11.3% (n=146/1293)

Engagement summaries (primary vs all participants)

Supplementary Table 5. Summary engagement statistics under the primary restriction (≤38 days) and in the full sample. Means in the full sample are influenced by the long right tail of recorded durations >38 days.

country	N	mean_days	sd_days	median_days	min_days	max_days	n_over_38	pct_over_38
Primary (<=38): Jamaica	1013	4.62	5.94	2.0	1	38	0	0.0
Primary (<=38): Malaysia	78	11.18	10.66	7.5	1	37	0	0.0
Primary (<=38): Philippines	75	3.45	5.29	2.0	0	36	0	0.0
Primary (<=38): Pooled	1166	4.99	6.54	2.0	0	38	0	0.0
All participants: Jamaica	1114	12.37	26.52	3.0	1	143	101	9.1
All participants: Malaysia	82	12.96	13.22	9.5	1	63	4	4.9
All participants: Philippines	97	21.52	35.70	3.0	0	136	22	22.7
All participants: Pooled	1293	13.09	26.78	3.0	0	143	127	9.8

Sensitivity analysis of alternative minimal-exposure thresholds

Supplementary Table 6. Percentage of participants below alternative completion thresholds, by country and pooled sample.

country	threshold_label	N_nonmissing	n_below	pct_below	formatted
Jamaica	<10%	1114	912	81.86715	81.9% (n=912/1114)
Jamaica	<15%	1114	998	89.58707	89.6% (n=998/1114)
Jamaica	<20%	1114	1043	93.62657	93.6% (n=1043/1114)
Jamaica	<25%	1114	1071	96.14004	96.1% (n=1071/1114)
Jamaica	<33%	1114	1096	98.38420	98.4% (n=1096/1114)
Jamaica	<50%	1114	1109	99.55117	99.6% (n=1109/1114)
Malaysia	<10%	82	41	50.00000	50.0% (n=41/82)
Malaysia	<15%	82	47	57.31707	57.3% (n=47/82)
Malaysia	<20%	82	52	63.41463	63.4% (n=52/82)
Malaysia	<25%	82	58	70.73171	70.7% (n=58/82)
Malaysia	<33%	82	67	81.70732	81.7% (n=67/82)
Malaysia	<50%	82	74	90.24390	90.2% (n=74/82)
Philippines	<10%	97	74	76.28866	76.3% (n=74/97)
Philippines	<15%	97	80	82.47423	82.5% (n=80/97)
Philippines	<20%	97	83	85.56701	85.6% (n=83/97)
Philippines	<25%	97	84	86.59794	86.6% (n=84/97)
Philippines	<33%	97	89	91.75258	91.8% (n=89/97)
Philippines	<50%	97	92	94.84536	94.8% (n=92/97)
Pooled	<10%	1293	1027	79.42769	79.4% (n=1027/1293)
Pooled	<15%	1293	1125	87.00696	87.0% (n=1125/1293)
Pooled	<20%	1293	1178	91.10596	91.1% (n=1178/1293)
Pooled	<25%	1293	1213	93.81284	93.8% (n=1213/1293)
Pooled	<33%	1293	1252	96.82908	96.8% (n=1252/1293)
Pooled	<50%	1293	1275	98.60789	98.6% (n=1275/1293)

Supplementary Table 7. Sensitivity of the minimal-exposure finding to alternative completion thresholds.

country	<10%	<15%	<20%	<25%	<33%	<50%
Jamaica	81.9% (n=912/1114)	89.6% (n=998/1114)	93.6% (n=1043/1114)	96.1% (n=1071/1114)	98.4% (n=1096/1114)	99.6% (n=1109/1114)
Malaysia	50.0% (n=41/82)	57.3% (n=47/82)	63.4% (n=52/82)	70.7% (n=58/82)	81.7% (n=67/82)	90.2% (n=74/82)
Philippines	76.3% (n=74/97)	82.5% (n=80/97)	85.6% (n=83/97)	86.6% (n=84/97)	91.8% (n=89/97)	94.8% (n=92/97)
Pooled	79.4% (n=1027/1293)	87.0% (n=1125/1293)	91.1% (n=1178/1293)	93.8% (n=1213/1293)	96.8% (n=1252/1293)	98.6% (n=1275/1293)

```
## Across the pooled sample, the proportion of participants below alternative completion thresholds was as follow
s: <10%: 79.4% (n=1027/1293); <15%: 87.0% (n=1125/1293); <20%: 91.1% (n=1178/1293); <25%: 93.8% (n=1213/1293); <3
3%: 96.8% (n=1252/1293); <50%: 98.6% (n=1275/1293).
```

```
## Country-specific proportions below each completion threshold were as follows: Jamaica (<10%: 81.9% (n=912/111
4); <15%: 89.6% (n=998/1114); <20%: 93.6% (n=1043/1114); <25%: 96.1% (n=1071/1114); <33%: 98.4% (n=1096/1114); <5
0%: 99.6% (n=1109/1114)) | Malaysia (<10%: 50.0% (n=41/82); <15%: 57.3% (n=47/82); <20%: 63.4% (n=52/82); <25%: 7
0.7% (n=58/82); <33%: 81.7% (n=67/82); <50%: 90.2% (n=74/82)) | Philippines (<10%: 76.3% (n=74/97); <15%: 82.5% (
n=80/97); <20%: 85.6% (n=83/97); <25%: 86.6% (n=84/97); <33%: 91.8% (n=89/97); <50%: 94.8% (n=92/97)).
```

Supplementary Table 8. Sensitivity of the minimal-exposure finding to alternative completion thresholds, by country and pooled sample.

country	<10%	<15%	<20%	<25%	<33%	<50%
Jamaica	81.9% (n=912/1114)	89.6% (n=998/1114)	93.6% (n=1043/1114)	96.1% (n=1071/1114)	98.4% (n=1096/1114)	99.6% (n=1109/1114)
Malaysia	50.0% (n=41/82)	57.3% (n=47/82)	63.4% (n=52/82)	70.7% (n=58/82)	81.7% (n=67/82)	90.2% (n=74/82)
Philippines	76.3% (n=74/97)	82.5% (n=80/97)	85.6% (n=83/97)	86.6% (n=84/97)	91.8% (n=89/97)	94.8% (n=92/97)
Pooled	79.4% (n=1027/1293)	87.0% (n=1125/1293)	91.1% (n=1178/1293)	93.8% (n=1213/1293)	96.8% (n=1252/1293)	98.6% (n=1275/1293)

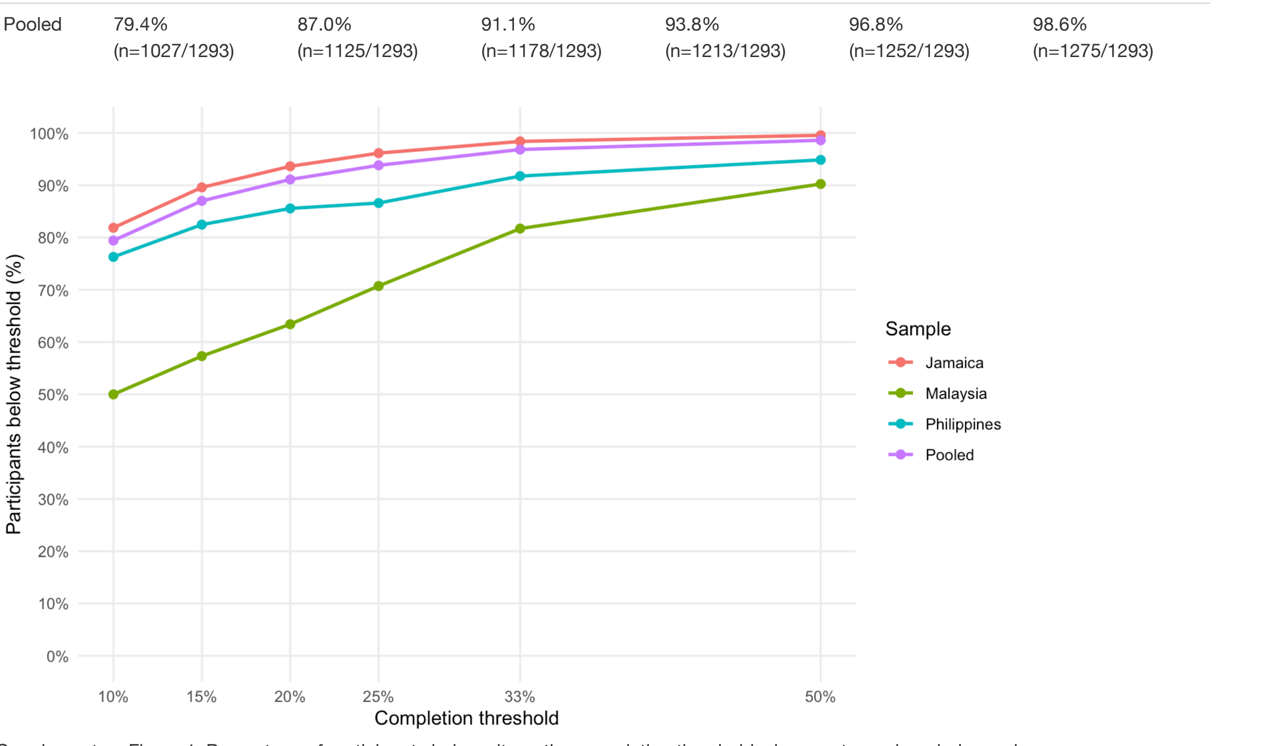

Supplementary Figure 1. Percentage of participants below alternative completion thresholds, by country and pooled sample.

Interpretation

The primary analyses, restricted to participants whose recorded time in programme fell within the planned 38-day intervention window, were designed to characterise retention and exposure to the intended programme. The capped-duration sensitivity analysis retained all enrolled participants while preventing late re-contact outside the planned intervention period from being misinterpreted as continuous retention. Across both approaches, the overall pattern of steep early attrition was unchanged.

The additional threshold sensitivity analysis showed that the inference of minimal exposure was not dependent on the specific <25% completion cutpoint used in the main manuscript. Across the pooled sample, 79.4% of participants completed less than 10% of available content, 87.0% less than 15%, 91.1% less than 20%, 93.8% less than 25%, 96.8% less than 33%, and 98.6% less than 50%. This indicates that the conclusion that most participants received only a small minority of available intervention content was robust across both more stringent and more lenient thresholds.

The same qualitative pattern was observed across countries. Although the exact percentages varied by site, the country-specific tables and Supplementary Figure 1 show that the large majority of participants in each implementation completed only a small fraction of the available programme content. Taken together, these findings support the interpretation that limited exposure was a general feature of the pilot implementations rather than an artefact of a single analytic decision about threshold selection or treatment of late re-contact.

For transparency, we note that if all participants are included and time in programme is calculated purely as the span between first and last interaction without capping, apparent retention at later timepoints can increase because late re-contact is counted as continued retention. We therefore do not interpret uncapped estimates as sustained engagement with the intended 38-day programme dose.

Conclusion

A subset of participants had recorded interactions beyond the planned 38-day programme period. Because time in programme is derived from the span between first and last interaction, late re-contact can inflate apparent retention at later timepoints if treated as continuous participation. When late re-contact is handled conservatively by capping time in programme at the planned duration, conclusions regarding early attrition and limited exposure within the intended programme window remain aligned with the primary analyses.
